# Interventions to promote healthy lifestyle behaviors in children and adolescents in summer day camps: a scoping review

**DOI:** 10.1186/s12889-023-15521-1

**Published:** 2023-04-26

**Authors:** David Larose, Melvin Chih-Shing Chen, Shirin Panahi, Jennifer Yessis, Angelo Tremblay, Vicky Drapeau

**Affiliations:** 1 Department of Kinesiology, Université Laval, Québec, G1V 0A6 Canada; 2 Quebec Heart and Lung Institute Research Center, Université Laval, Québec, G1V 0A6 Canada; 3 Centre Nutrition, santé et société (NUTRISS), Institute of Nutrition and Functional Foods (INAF), Université Laval, Québec, G1V 0A6 Canada; 4 Centre de recherche interuniversitaire sur la formation et la profession enseignante (CRIFPE), Université Laval, Québec, G1V 0A6 Canada; 5 School of Public Health Sciences, University of Waterloo, Waterloo, N2L 3G1 Canada; 6 Department of Physical Education, Université Laval, Québec, G1V 0A6 Canada

**Keywords:** Summer Day Camps, Physical activity, Sedentary behaviors, Healthy eating, Healthy lifestyle behaviors, Children, Adolescents

## Abstract

**Background:**

Children and adolescents have suboptimal physical activity and eating habits during summer breaks. Unlike the school setting, there is little evidence on interventions to promote healthy lifestyle behaviors in Summer Day Camps (SDCs).

**Methods:**

The aim of this scoping review was to examine physical activity, healthy eating, and sedentary behavior interventions in the SDCs. A systematic search on four platforms (EBSCOhost, MEDLINE, EMBASE, and Web of Science) was performed in May 2021 and was updated in June 2022. Studies related to promoting healthy behaviors, physical activity, sedentary behaviors and/or healthy eating among campers aged 6 to 16 in Summer Day Camps were retained. The protocol and writing of the scoping review were done according to the guidelines of the “Preferred Reporting Items for Systematic reviews and Meta-Analyses extension for scoping reviews (PRISMA-ScR)”.

**Results:**

Most interventions had a positive effect on the behavioral determinants or the behaviors themselves (i.e., physical activity, sedentary behaviors, or healthy eating). Involving counsellors and parents, setting camp goals, gardening, and education are all relevant strategies in promoting healthy lifestyle behaviors in SDCs.

**Conclusions:**

Since only one intervention directly targeted sedentary behaviors, it should strongly be considered for inclusion in future studies. In addition, more long-term and experimental studies are needed to establish cause-and-effect relationships between healthy behavior interventions in SDCs and behaviors of children and young adolescents.

**Supplementary Information:**

The online version contains supplementary material available at 10.1186/s12889-023-15521-1.

## Introduction

The promotion of healthy lifestyle behaviors such as physical activity and healthy eating is a priority among children and adolescents, especially since the healthy behaviors developed early in life persist into adulthood [1]. Regular physical activity and healthy eating habits can improve musculoskeletal health, decrease symptoms of anxiety, depression, and risk of chronic disease, and promote academic performance [2]. While there appears to be difficulty meeting recommendations for both physical activity and nutrition during school [3], one would expect children and young adolescents to be more active during summer breaks and have better eating habits given the increased supply of fresh fruits and vegetables. However, physical activity during this period is rather marked by weight gain and a decline in fitness [4, 5] partially explained by a lack of organization of activities and support by adults regularly offered through institutions such as school or extracurricular programs [6]. Summer can also present an open and autonomous environment for children that can negatively influence their eating habits [7]. Indeed, during summer breaks, many children and adolescents do not accumulate 60 min of moderate-to-vigorous physical activity (MVPA) per day [8] as recommended by the most recent 24-h guidelines [9]. They also have low consumption of vegetables, and high consumption of sugar-sweetened beverages (SSB) [8] contrary to what is recommended by the 2019 Canada's Food Guide [10].

Considered a setting for organized activities for children during summer breaks, Summer Day Camps (SDCs) appear to be a good solution to the problem of organization and supervision of activities. However, even if SDCs address the lack of organization and support, they do not necessarily offer opportunities for children and young adolescents to be physically active for at least 60 min per day. Observation tools in SDCs showed that only 38% of weekly plans were devoted to physical activity and that only 19% and 18% of children and adolescents participating in physical activities organized by the camps were engaged in moderate or vigorous physical activity, respectively [11]. In addition, an observational study among campers showed that only 20% and 4% of lunch boxes contained a fruit and a vegetable, respectively [12]. This study also concluded that 47% of campers had brought non-100% juice and 4% had soft drinks in their lunch boxes, indicating a large intake of SSB [12]. The fluid intake of campers also seems inadequate considering that many of them drank no beverages at all at any of the meals across the entire day [13]. Children and adolescents, therefore, have suboptimal physical activity and eating habits, especially during summer breaks. To date, there is little evidence on interventions to promote healthy lifestyle behaviors in SDCs to improve physical activity, sedentary behaviors, and eating habits of campers. The overall aim of this scoping review is to describe the interventions promoting healthy lifestyle behaviors in children and adolescents, particularly those that involve physical activity, sedentary behaviors, and healthy eating in SDCs settings in order to guide further interventions.

## Methods

### Design

This study used the scoping literature review design described by Arksey and O’Malley [14] to explore the available literature, guide future interventions, and pave the way for further systematic reviews based on gaps in this research area. According to this design, the quality of the studies was not assessed and does not constitute a condition for rejection [14]. This scoping review conforms to the “Preferred Reporting Items for Systematic reviews and Meta-Analyses extension for scoping reviews (PRISMA-ScR)” [15].

### Research questions

The purpose of this scoping review is 1) to map the interventions promoting healthy lifestyle behaviors, particularly those that involve physical activity, sedentary behaviors, and healthy eating in SDC settings, and 2) to describe the effects on campers and identify gaps and promising strategies for future interventions.

### Identifying relevant studies

A literature search was performed with the support of an experienced librarian (Marie Denise Lavoie) in May 2021 and was updated in June 2022 to capture the studies. The following platforms were accessed: SPORTDiscus, CHILD DEVELOPMENT & ADOLESCENT STUDIES, ERIC, EDUCATION SOURCE, MEDLINE, EMBASE, and Web of Science [See Additional file 1 for detailed search methodology].

A targeted search based on the concepts "promotion of healthy lifestyles and/or health" OR "physical activity" OR "eating habits" AND "Summer Day Camp" was carried out, with variants adapted for each database, if applicable. To be included in the review, studies must a) be related to the promotion of healthy behaviors; b) be related to physical activity, sport, exercise, outdoor games, sedentary behavior and/or diet, eating habits, nutrition, and healthy eating; c) be in Summer Day Camps of varying lengths to which access is public and not private; and d) include a sample of children and young adolescents aged 6 to 16 years. The following items have been excluded: a) studies related to a setting that includes camping; b) studies related to a framework specific to public holidays; c) studies related to a framework that includes school environments; d) studies related to a framework that includes specific food consumption; e) studies related to a framework that includes eating disorders; f) studies in which the sample includes specific conditions (e.g., weight loss camp, those diagnosed with severe mental illness or physical disability); g) unpublished studies or non-intervention studies; and h) articles that were not in English or French.

### Study selection

All references were imported into EndNote 20 software (Clarivate Analytics, Philadelphia, United States) and duplicates were removed using Covidence (i.e., a screening and data extraction tool). The remaining titles and abstracts were screened by two independent reviewers (DL, MCSC) using predetermined inclusion/exclusion criteria. Full texts were independently reviewed against inclusion/exclusion criteria. Discrepancies were resolved by a third independent reviewer (SP). Consensus was reached for all included articles.

### Data charting

A data extraction table was created in Microsoft Excel by the research team by identifying different variables consistent with the objective of the scoping review. Then, two reviewers independently extracted information from relevant articles and charted the data using the same extraction table. The key variables included citation, research question, framework, sample population (i.e., the number of participants who were considered for the statistical analysis, not the complete sample), research design, data collection methods and measures, counsellors’ implication, intervention, and main results based on campers and environment (i.e., counsellors, parents, SDCs setting).

### Collecting, summarizing, and reporting the results

**Fig. 1:**
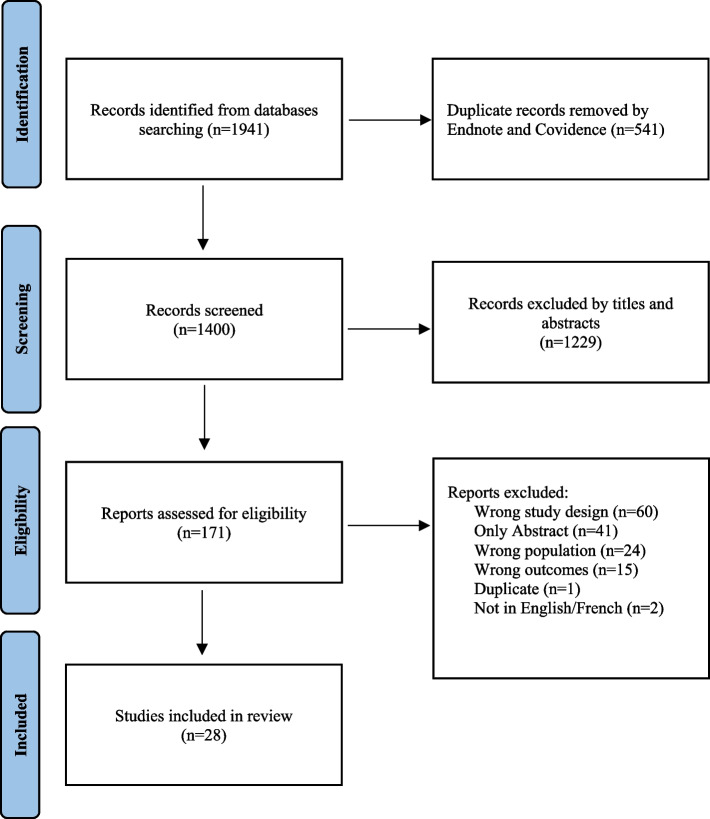
PRISMA flow diagram of study selection process

The PRISMA diagram was used to illustrate the review process and specifies the number of articles rejected for each of the main reasons for exclusion (Fig. 1). The data from the approved articles were synthesized and classified according to the variables previously presented in an excel document.

## Results

### Study characteristics

A total of 1941 articles were initially identified and imported into the Endnote and then Covidence software. After removing duplicates (*n* = 541), a total of 1400 articles were screened by title and abstract, 171 articles were full text filtered, and 28 studies met our eligibility criteria. The main reasons for exclusion were study design (e.g., there was no intervention), article availability (e.g., some were impossible to find while many were only abstracts of published conferences), participants (e.g., children had specific health problems such as diabetes or vision problems), study outcomes were not relevant (e.g., weight or waist circumference) or the language (i.e., the article was not in English or French). Of the articles selected, eight interventions specifically targeted the promotion of physical activity, fourteen interventions focused on healthy eating, five targeted both physical activity and healthy eating, and one intervention was specific to physical activity, sedentary behaviors, and healthy eating. Among all these interventions, only five are not based on precise theoretical frameworks [16–20]. To measure the effects of these interventions, seven studies used an experimental design, eighteen studies used a quasi-experimental design, two studies used a mixed methods design, and one study used a qualitative design. Sixteen of the studies using a quasi-experimental design did not have a control group and seventeen did not randomize their sample. Most of these studies used a pre- and post-intervention design and seven studies had a follow-up. All the studies were cross-sectional, except for four longitudinal studies. Three of the cross-sectional studies measured the effects of the intervention over several years (i.e., more than one year), but with different groups of campers (Table 1).

**Table 1:** Description and design of included studies

**References**	**Target**	**Aims**	**Research design (protocol)**	**Framework (Theoretical frame)**	**Data Collection, Methods & Measures**
Anderson-Butcher et al., 2019 [16]	Physical activity	To examine the influence of support from program staff and parents on fitness, self-efficacy, and health and fitness intentions of disadvantaged, urban youth participating in the LiFEsports program	• Quasi-experimental • ∅ Control group • ∅ Randomized • Measurement: Pre (day 0)—Post • Involvement of counsellors	NA	Administered: • Fitness test (aerobic cardiovascular endurance) Self-reported: • Questionnaire (PA self-efficacy + health and fitness intentions + staff and parent support for health and fitness)
Baranowski et al., 2003 [21]	• Physical activity • Healthy eating	To test the "The Girls health Enrichment Multi-site Studies (GEMS) Fun, Food, and Fitness Project (FFFP)" intervention over 12 weeks (intervention process measures and trends in key measurements, including body mass index, diet, PA, and psycho-social measures)	• Experimental • Control group • Randomized • Measurement: Pre (week 0) – Post (week 4)—Follow-up (week 16)	SCT	Administered: • Accelerometer/pedometer Self-reported: • 24-h dietary recalls • Questionnaire (GEMS activity questionnaire (i.e., 24-h PA recall) (PA + PA preference + sweetened beverage preference))
Beets et al., 2007 [22]	Healthy eating	To increase the number of times campers prepared meals at home, to improve their attitudes, self-efficacy, behavioral expectancies, knowledge, perceived cooking ability, and perceptions of parents' worry regarding cooking	• Quasi-experimental • ∅ Control group • ∅ Randomized • Measurement: Pre (week 0)—Post (week 1)	Experiential learning framework	Self-reported: • Questionnaire (cooking behaviors + psychosocial constructs related to preparing food (i.e., attitudes toward cooking, self-efficacy, behavioral expectancies, knowledge, perceived cooking ability, and perceptions that parents worry about cooking))
Beets et al., 2014 [23]	Healthy eating	To develop and evaluate an innovative healthy eating intervention, called the healthy lunchbox challenge (HLC), designed to increase the amount of FV and 100% fruit juice children and staff bring to SDCs and to align staff behaviors with those called for in the NAA HEPA standards	• Quasi-experimental • ∅ Control group • Randomized • Measurement: Pre (summer 2011) – Post (summers 2012 and 2013) • Involvement of counsellors	• BCT • Goal-setting theory	Administered: • Observations of campers and counsellors (all food and beverage items brought to SDCs by children and staff + SOSPAN (Staff Promotion of Activity and Nutrition))
Bohnert et al., 2017 [24]	• Physical activity • Healthy eating	To examine whether a structured summer camp setting benefits children's PA and dietary intake	• Quasi-experimental • ∅ Control group • ∅ Randomized • Measurement: Pre (week 1)—Post (week 4) • Data collected over 3 summers	Bio-ecological perspective	Administered: • Accelerometer/pedometer Self-reported: • 24-h dietary recalls
Brazendale et al., 2020 [25]	Physical activity	To evaluate a multi-component intervention to increase the percentage of children meeting 60 min/d of MVPA	• Quasi-experimental • Control group • ∅ Randomized • Measurement: Pre (summer 2015) – Post (summers 2016 and 2017) – Follow-up (summer 2018) • Involvement of counsellors	• STEPs • TEO	Administered: • Accelerometer/pedometer • Observation (SOPLAY (Play and Leisure Activities (campers)) + SOSPAN (Staff Promotion of Activity and Nutrition))
Condrasky et al., 2015 [26]	Healthy eating	To explore the relationship between nutrition knowledge, cooking skills, and confidence and motivation for early adolescents to make healthier food choices	• Quasi-experimental • ∅ Control group • ∅ Randomized • Measurement: Pre (week 0)—Post (week 1 or 4)	SCT	Self-reported: • Questionnaire (confidence & motivation + Let's Eat Healthy (nutrition knowledge) + cooking skills)
Ehrenberg et al., 2019 [27]	Healthy eating	To test whether children’s preferences for target fruits and vegetables increased following repeated taste exposures to them through hands-on cooking in a community setting	• Quasi-experimental • ∅ Control group • ∅ Randomized • Measurement: Pre (week 0)—Post (Midpoint and 6-week)	Repeated exposure approach	Self-reported: • Questionnaire (familiarity, liking, preferences for individual study foods)
Gachupin et al., 2019 [17]	• Physical activity • Healthy eating	To describe the impact of the program, Healthy 2B Me camp, on camp participants and parents	• Quasi-experimental • ∅ Control group • ∅ Randomized • Measurement: Pre (week 0)—Post (week 2 (2013) and week 3 (2014- 2016))—Follow-up (90-days (parents)) • Data collected over 4 summers	NA	Self-reported: • Questionnaire (knowledge, attitudes, behavior towards healthy eating + PA)
Harmon et al., 2015 [28]	Healthy eating	To explore children’s involvement in meal preparation at home, from the perspectives of camp participants and their parents, and to examine changes in the children’s attitudes and self-efficacy related to cooking to inform future culinary program development and implementation	• Quasi-experimental + Qualitative (Mixed-methods) • ∅ Control group • ∅ Randomized • Measurement: Pre (week 1 (Quantitative only))—Post (week 4 (Quantitative + Qualitative))	Experiential learning framework	Administered: • Interview Self-reported: • Questionnaire (involvement in family meal preparation + attitudes + self-efficacy related to cooking)
Heim et al., 2009 [29]	Healthy eating	To evaluate the Delicious and Nutritious Garden, a 12-week garden-based nutrition intervention	• Quasi-experimental • ∅ Control group • ∅ Randomized • Measurement: Pre (before participating in garden-based activities)—Post (week 12) • Involvement of counsellors	• Experiential learning approach • SCT	Self-reported: • Questionnaire (enjoyment for each intervention activity + FV exposure + preferences + self efficacy + asking behavior + home availability)
Jacob et al., 2020 [20]	Healthy eating	To measure the influence of the Chefs in Action program (3 cooking workshops) on cooking skills, nutrition knowledge, and attitudes towards healthy eating in children attending SDCs and compare it with a single cooking workshop	• Experimental • Control group (Random assignment to an intervention or 1 of 3 comparison groups) • Randomized • Measurement: Pre (week 0)—Post (week 3)	NA	Administered: • Observation (cooking skills) Self-reported: • Questionnaire (nutrition knowledge + attitudes towards healthy eating)
Kimiecik et al., 2021 [18]	Physical activity	To measure (a) what perceived program mechanisms and design components influence participation in a sport-based PYD program; (b) what perceived health and well-being outcomes are influenced by participation in a sport-based PYD program; (c) if there are differences in girls’ holistic health and well-being outcomes following participation in a sport based PYD program	• Quasi-experimental + Qualitative (Mixed-methods) • ∅ Control group • ∅ Randomized • Measurement: Pre (week 1 (Quantitative only))—Post (week 4 (Quantitative + Qualitative)) • Involvement of counsellors	NA	Administered: • Interview Self-reported: • Questionnaire (The Healthy Lifestyle Behavior Scale (perceptions about their health behaviors) + The Social Competence Scale (perceived social competence)
Lawman et al., 2019 [30]	Healthy eating	To determine the effectiveness of the “Hydrate Philly” multi-level intervention to increase water access and appeal in community recreation centers in (a) increasing center-level water intake as measured by water flow meters, and (b) decreasing the purchase of outside beverages as measured by observations of youth visiting recreation centers	• Experimental • Control group • Randomized (matched pair) • Measurement: Pre (week 0)—Post (3–5 months)—follow-up (7–9 months) • Involvement of counsellors	• The social ecological model • SCT	Administered: • Observation (water consumption + tally of reusable water bottles + sugary beverages + bottled water + weight of the day’s trash) • Water consumption (objective water flow meters) Self-reported: • Questionnaire (staff SSB consumption (BEVQ- 15))
Mabary-Olsen et al., 2015 [31]	Healthy eating	To improve self-efficacy and health behaviors related to nutrition and PA through experiential learning activities at summer camps	• Experimental • Control group • Randomized • Measurement: Pre (week 0)—Post (week 3)—Follow-up (6-months)	• Experiential learning approach • Immersion programming	Self-reported: • Questionnaire (FV knowledge + FV preferences + self-efficacy for FV intake + home food environment behavior change + dietary intake (food frequency questionnaire))
Maxwell et al., 2018 [32]	Healthy eating	To assess the feasibility of adapting the evidence-based intervention and implementing it in the busy environment of a YMCA SDC, and to explore the short-term impact of the program on children’s liking of initially disliked vegetables and on their willingness to try new foods	• Quasi-experimental • ∅ Control group • Randomized (NA) • Measurement: Pre (week 0)—Post (week 2)—follow-up (week 4)	Self-determination theory	Self-reported: • Questionnaire (availability of FV + consumption of FV yesterday + liking of 12 vegetables and 8 fruits)
Murad et al., 2021 [33]	Healthy eating	To describe a virtual kids’ cooking camp and evaluates how well it served the campers and the university students who developed it	• Quasi-experimental • ∅ Control group • Randomized (NA) • Measurement: Pre (week 0)—Post (week 1)	SCT	Self-reported: • Questionnaire (food literacy (TFLAC) + confidence in making sustainable food choices)
Reverter-Massia et al., 2012 [19]	• Physical activity • Healthy eating	To quantify the long- and short-term effectiveness of healthy habits of children involved in an educational intervention program consisting of a presentation and explanation of the “Healthy lifestyle guide pyramid” on nutritional habits, daily activities, and health	• Experimental • Control group • Randomized • Measurement: Pre (week 0)—Post (week 1)—follow-up (2-months)	NA	Self-reported: • Questionnaire (nutritional habits + daily activities + health)
Seal et Seal 2011 [34]	• Physical activity • Healthy eating	To test the short-term effects of the Wellness Summer Camp (WSC) program on changes in children's knowledge of healthy foods and healthy snacks, physical activity and eating behaviors, and self-perception of competence in school-age children	• Quasi-experimental • ∅ Control group • ∅ Randomized • Measurement: Pre (Day 1)—Post (Day 10) • Involvement of counsellors	• Age- appropriate developmental theory • SCT	Self-reported: • Questionnaire (health behavior + self-perception of competence)
Tauriello et al., 2020 [35]	Healthy eating	To examine whether pairing a non-food stimulus with target vegetables increases children's vegetable acceptance and whether effects exceed those of repeated exposure	• Experimental • Control group • Randomized • Measurement: Pre (week 1)—Post (week 6)	• Associative conditioning • Positive peer context	Self-reported: • Questionnaire (vegetable familiarity, liking, and preference + positive peer context liking)
Tilley et al., 2014 [36]	Healthy eating	To describe the development and evaluation of the Healthy Lunchbox Challenge (HLC), an innovative, theory and incentive-based program to influence the number of fruits, vegetables, and water brought to SDC by children. As a secondary outcome, the HLC also targeted the foods and beverages brought by staff	• Quasi-experimental • ∅ Control group • ∅ Randomized • Measurement: Pre (summer 2011) – Post (summer 2012) • Involvement of counsellors	• BCT • Goal-setting theory	Administered: • Observations of campers and counsellors (all food and beverage items brought to SDCs by children and staff)
Warner et al., 2021 [37]	Physical activity	To explore whether the physical literacy levels of youth facing barriers, aged 6–10, could be increased through the implementation of a 2-week day camp-style program	• Quasi-Experimental • ∅ Control group • ∅ Randomized • Measurement: Pre (Day 1)—Post	• Maple Leaf Sport and Entertainment LaunchPad's Theory of Change • Sport for Development • Fundamental Movement Skills	Administered: • Fitness test (Fundamental of Movement Skills (FMS)) (i.e., environment and self-perception of physical competence) Self-reported: • Questionnaire (PLAYself Physical Activity Assessment for Youth (i.e., physical literacy (competence, confidence, motivation, and knowledge)))
Weaver, Beets, Saunders et al., 2014 [38]	Physical activity	To describe the development and first-year outcome evaluation of competency-based professional development training on staff engagement in HEPA promoting behaviors and the impact on children's activity levels	• Quasi-experimental • ∅ Control group • ∅ Randomized • Measurement: Pre (2011)—Post (2012) • Involvement of counsellors	• The 5Ms training model • LET US Play principles	Administered: • Observation (SOPLAY (Play and Leisure Activities (campers)) + SOSPAN (Staff Promotion of Activity and Nutrition))
Weaver, Beets, Turner-McGrievy et al., 2014 [39]	Physical activity	To describe a three-year partnership between the University and local YMCA to provide competency-based professional development training and the impact of the training on children's activity levels in participating SDCs	• Quasi-experimental • ∅ Control group • ∅ Randomized • Measurement: Pre (2011)—Post (2012 and 2013) • Involvement of counsellors	• The 5Ms training model • LET US Play principles	Administered: • Observation (SOPLAY (Play and Leisure Activities (campers)) + SOSPAN (Staff Promotion of Activity and Nutrition))
Weaver et al., 2017 [40]	Physical activity	To evaluate an intervention designed to increase the % of children meeting the MVPA guideline	• Quasi-experimental • Control group • ∅ Randomized • Measurement: Pre (summer 2015) – Post (summer 2016) • Involvement of counsellors	TEO	Administered: • Accelerometer/pedometer • Observation (SOSPAN (Staff Promotion of Activity and Nutrition)) Self-reported: • Schedule
Werner et al., 2012 [41]	• Physical activity • Healthy eating • Sedentary behavior	To evaluate an intergenerational childhood obesity prevention project called Active Generations	• Experimental • Control group • Randomized • Measurement: Pre (day 1)—Post (last day)	Intergenerational, evidence-based programming	Self-reported: • Questionnaire (knowledge + attitudes + behaviors (PA, nutrition, and screen time))
Williams et al., 2019 [42]	Healthy eating	To evaluate the feasibility of implementing a cooking curriculum into a summer day camp to determine its reception and explore the potential of reach at home	• Qualitative • Measurement: Only post (5–6 weeks)	Experiential-learning approach	Administered: • Qualitative interview
Wilson et al., 2017 [43]	Physical activity	To assess the impact of three different goal-setting programs with pedometers on children’s physical activity and enjoyment in a day camp setting	• Quasi-experimental • ∅ Control group • ∅ Randomized • Measurement: Pre (Baseline, 1 week prior)—Post (3 subsequent weeks) • Involvement of counsellors	Goal-setting theory	Administered: • Accelerometer/pedometer Self-reported: • Funometers (PA enjoyment)

*BCT* Behavioral choice theory

*SCT* Social Cognitive Theory

*STEPs* Strategies To Enhance Practices

*TEO* Theory of Expanded, Extended and Enhanced Opportunities

### Measurement of physical activity, sedentary behaviors, and healthy eating

Of the fourteen studies that assessed physical activity, twelve of them measured the effects of promoting physical activity on the physical activity of children and young adolescents [17–19, 21, 24, 25, 34, 38–41, 43], while the other two only measured the effects of the interventions on determinants of physical activity. Physical activity (PA) was measured using accelerometers/pedometers in five studies [21, 24, 25, 40, 43], using questionnaires (i.e., self-reported data) in six studies [17–19, 21, 34, 41], and using a validated time-sampling observation tool (SOPLAY) in three studies [25, 38, 39]. For the evaluation of the determinants of physical activity using questionnaires, one study measured intention and self-efficacy [16], one study measured physical literacy and barriers towards physical activity [37], two studies measured knowledge and attitudes towards physical activity [17, 41], and one study measured enjoyment [43].

Among the twenty articles that evaluated interventions targeting the promotion of healthy eating, twelve studies measured eating habits [19, 21, 23, 24, 26, 28, 30–32, 34, 36, 41]. Eight studies used questionnaires [19, 26, 28, 30–32, 34, 41], three used observations [23, 30, 36], and two of them used 24-h dietary recalls [21, 24]. The main determinants of healthy eating measured were food preferences, liking, knowledge, self-efficacy, motivation, environment, exposure, availability, cooking skills, cooking behavior, involvement in family meals, and attitudes [17, 20–22, 26–29, 31–35, 41, 42].

A few studies have measured the sedentary behaviors of campers, either with accelerometers/pedometers [24], observations (SOPLAY) [38, 39] or with questionnaires (i.e., screen time) [19, 41]. Nonetheless, only one intervention targeted sedentary behaviors [41].

### Effect of Summer Day Camp interventions targeting physical activity or sedentary behaviors

Eight of the twelve studies that measured physical activity observed increases across different measures. Studies by Bohnert et al. [24], Gachupin et al. [17], Weaver, Beets, Saunders et al. [38], Weaver, Beets, Turner-McGrievy et al. [39], and Weaver et al. [40] measured increases in moderate-to-vigorous physical activity using different tools (i.e., accelerometer/pedometer, questionnaire, and SOPLAY). Kimiecik et al. [18] observed differences in how campers perceived their behaviors to be healthier after the summer. Reverter-Masia et al. [19] observed an increase in short- and long-term physical activity after the intervention and Wilson et al. [43] measured a greater number of steps taken by campers during the SDCs (Table 2).

**Table 2:** Population, intervention, and results of included studies

**References**	**Sample Population**	**Intervention**	**Main Results or Key Findings**	
			**For campers**	**Environment (counsellors, parents, SDCs setting)**
Anderson-Butcher et al., 2019 [16]	• 375 campers: mean age of 11.3 years old • 1 camp	• The LiFEsports Initiative (PA) • 19 days • Enhance self-control, effort, teamwork, and social responsibility (S.E.T.S.) • 15 h of social competence curriculum focused on S.E.T.S., five hours of sports instruction, and five hours of a healthy lifestyle behaviors curriculum	• Aerobic Cardiovascular Endurance: ⬆ • Physical activity self-efficacy scores: ⬆ • Support for health and fitness intentions from parents and staff: ⬆ (Physical activity self- efficacy and health and fitness intentions)	• PA self-efficacy: Parent and staff support, and Pre-camp PA self- efficacy were significant and positive predictors of the post- camp score. The positive effect of support from staff tended to increase in magnitude as the degree of support from the parents increased
Baranowski et al., 2003 [21]	• 35 campers: mean age of 8 years old • 1 camp	• Fun, Food, and Fitness Project (PA + HE) • 12-week intervention (4-week in camp and 8-week Internet (web programs)) • Increase PA, enhance social support, involve the parent, increase camper exposure to PA + provide them with a pedometer to self-monitor PA + encourage to consume more FV and 100% fruit juice (FJV), and drink water • The camp program: buddy groups, camp cheers, problem solving, dance, educational games, snack preparation, and goal setting • The web programs: a comic book and PA goals, problem solving, review of attainment of previous week’s goal, a photo album of girls from the camp, an “ask the expert” feature, and links to various websites of interest to girls	• PA measures: ∅ • Total calories: ⬇ • % calories from fat: ⬇ • Consumption of FJV: ⬆ • Servings of sweetened beverages: ⬇ • Servings of water: ⬆ • PA preference: ∅ • Sweetened beverages preference: ∅	NA
Beets et al., 2007 [22]	• 17 campers • 1 camp	• The Culinary Camp Summer Cooking Program (HE) • Eight days, with sessions lasting four hours • Emphasized development of autonomy, active participation in the selection, preparation, and consumption of regional and culturally diverse food • The program involved the development of culinary skills and discussions regarding the types of foods, the difficulties encountered, and the modifications made to the recipe	• Cooking behaviors: ∅ • knowledge (nutrition): ⬆ • Perceived cooking ability: ⬆ • Negative attitudes: ⬇ (trend)	NA
Beets et al., 2014 [23]	• 550 campers: 6- 12 years old, mean age of 7.8 years old • 48 – 60 counsellors • 4 camps	• Healthy lunchbox challenge (HE) • 11-week schedule throughout the summer with parents enrolling their children in a camp for one week • One training at the beginning of each intervention summer (45 min) for SDC directors and staff (healthy eating promotion) • Support (weekly communications + resolve implementation errors) • A point system was developed where children could earn up points per day • Parents received HLC materials designed to influence decisions of foods/ beverages purchased for SDC	• FV consumption: ⬆ (by summer 2013) • Water consumption: ∅ • Unhealthy foods/beverages (soda/pop, non 100% juice, chips, and fast food): ⬇	• FV counsellors’ consumption: ∅ • Unhealthy foods/beverages (counsellors): ⬇ • Staff promoting healthy eating and educating children about healthy eating: ⬆ • Staff consuming inappropriate foods and drinks in front of campers: ⬆
Bohnert et al., 2017 [24]	• 64 campers: 10- 14 years old, mean age of 11.9 years old • 1 camp	• Girls in the Game (PA + HE) • Four weeks, six hours of structured activities each day (three 50-min morning sessions (i.e., two sports-based PA lessons and one health/leadership activity), a 40-min lunch break, 60 min of pool time, 45 min of team PA, as well as an additional 10-min snack break) • Each session provided instruction and PA through a variety of sports and fitness activities	• 5-min MVPA bouts/day: ⬆ (1.68) • Additional minutes/day spent in 5-min MVPA bouts: ⬆ • Sedentary time: ⬇ (2 h and 29 min/day) • Total calories and fat consumed: ∅ • Fruit consumption: ⬆ (1.19 servings/day) • Dairy consumption: ⬇ (0.75 servings/day) • Sweets and sugar-sweetened beverages consumption: ⬇ (trend)	NA
Brazendale et al., 2020 [25]	• 3524 campers: ≤ 12 years old • 20 camps	• Turn up the healthy eating and activity time (HEAT) (PA) • Duration of the program (NA), 10 SDCs received two summers of the PA intervention and 10 SDCs received a single summer (2017) • Camp leaders and staff receive training to expand, extend, and enhance PA opportunities (i.e., a single 90-min professional development training session and a 30-min discussion on strategies to address challenges observed with increasing children's PA) • Two on-site booster sessions (Walkthrough of the SDC and discussion to address challenges observed with increasing children’s MVPA)	• Intervention for 2 years versus 1 year: ∅ • Likelihood of meeting the 60 min/d MVPA: ∅ (boys or girls) • Girls and boys were 3.5 and 3.7 times more likely to meet the 60 min/d guidelines during intervention summers versus follow-up, respectively	• An average of 5 SDCs enhanced physical activity opportunities during intervention summers vs. baseline by increasing their LET US Play Index score • Comparing follow-up to baseline, 8 SDCs (4 immediate intervention, 4 delayed intervention) increased their LET US Play Index score
Condrasky et al., 2015 [26]	• 56 campers: 10- 14 years old • 2 camps	• The Cook Like a Chef program (HE) • Two comparable samples, 5 or 20 interactive culinary nutrition sessions • The 1-week model: demonstration and nutrition discussion + an hour of cook time + The campers tasted recipes of the day in a shared meal + a quick healthy snack demo at the end of each day • The 4-week model: a shared breakfast meal + a 10-min walk to the Family and Consumer Sciences foods lab + a nutrition lesson and a cooking demonstration + three hours of cooking time + shared lunch + a variety of physical activities and afternoon healthy snacks. The camp also included field trips to a Whole Foods Grocery Store and the Louisiana Food History Museum	• Nutrition knowledge, cooking skills, and motivation and confidence to prepare healthy meals and snacks: ⬆ (1- and 4-week models) • Food safety scores: ∅ (for or between the 1- and the 4-week camps) • Food nutrients and sources scores: ∅ (for the 1- and the 4-week camps)	NA
Ehrenberg et al., 2019 [27]	• 17 campers: 6–8 years old, mean age of 7.1 years old • 1 camp	• Mini-Chefs (HE) • Biweekly hands-on cooking program for six weeks • Children were exposed to each of the four target foods (bell peppers, tomatoes, cantaloupe, and nectarines) five times across nine different snacks that they made with the help of study staff and ate together as a class • During each exposure session, children worked together to follow the day’s recipe and assemble the snack, using child-safe knives to cut the fruits and vegetables. Once complete, each child was given a serving of the prepared snack, and children sat together at classroom tables to eat	• Preferences for target foods (tomatoes, bell peppers, cantaloupe, and nectarines): ⬆ • Preferences for target vegetables and target fruits separately: ∅ • Initial liking of the target foods did not predict whether or not children increased their preferences for them from pre-test to post-test	NA
Gachupin et al., 2019 [17]	• 187 campers: 7- 11 years old, mean age of 8.5 years old • 4 camps	• The Healthy 2B Me summer camp (PA + HE) • Eight hours, two-week-long (2013), or three week-long sessions (2014–2016) • Educate and empower through knowledge, attitude, and behavior changes + increase parental involvement in supporting healthy behaviors in their children • Focused on nutrition (ex. food labeling education, healthy guidelines, etc.), PA, hand washing, smoking, sun safety or kindness, lessons were interactive and interspersed with PA (60 min + per day)	• PA knowledge: ⬆ • Attitude toward PA: ⬆ • MVPA every day: ⬆ • Nutrition knowledge (FV serving sizes): ⬆ • Attitudes toward FV: ⬆	NA
Harmon et al., 2015 [28]	• 30 campers: 9- 12 years old • 20 completed qualitative interviews • 1 camp	• Culinary Skills Training (HE) • Four culinary skills training sessions (one hour, once per week) • Each session focused on teaching culinary skills (i.e., knife skills, measuring, safe handling of food, and types of cooking methods) and incorporating whole grains, fruits, and vegetables into each recipe via a hands-on approach • Participants were given take-home assignments (i.e., additional recipes to make at home and an evening meal journal for their parents to complete) and “coupons” to share with their parents	• Attitude: ⬆ (not significant) • Liking to cook and the belief that fruits and vegetables are important: ∅ • Perceived cooking skills and abilities: ⬆	• Meaningful changes were not seen in the food environment (questionnaire)
Heim et al., 2009 [29]	• 93 campers: 8- 11 years old, mean age of 9.7 years old • 1 camp	• The Delicious and Nutritious Garden (HE) • 12-week summer camp (children signed up for camp on a weekly basis) • Beans, beets, carrots, cabbage, cucumbers, eggplant, kohlrabi, leaf lettuce, okra, onions, peppers, radishes, strawberries, Swiss chard, summer squash, tomatoes, zucchini, and herbs were planted by children in the first and second weeks of the intervention. Children also learned to weed, observe, and harvest their garden. Garden-based activities included learning about the origins of food, plant parts, nutrient needs of humans and plants, environmental stewardship, MyPyramid for Kids, goal setting, and role-playing • The children prepared a dozen healthful snacks with produce from their garden, including two snacks for younger campers to promote peer modeling of fruit and vegetable intake. They all received a cookbook containing recipes for the FV they taste-tested and prepared throughout the intervention • Parents/primary caregivers were encouraged to improve FV availability and accessibility through weekly newsletters, recipes, and take-home activities	• Number of fruits and vegetables ever eaten: ⬆ • Vegetable preferences: ⬆ • Fruit preferences: ∅ (high) • Snack preferences: ∅ • Self-efficacy to consume FV: ∅ • Child asking behavior: ⬆	NA
Jacob et al., 2020 [20]	• 101 campers: 8- 12 years old • 2 camps	• The Chefs in Action program (HE) • One 30-min workshop was held per week for 3 weeks • Promote the pleasure and importance of healthy eating, support the development of cooking skills, and expose children to a variety of foods • A demonstration of the recipe was first performed. The demonstration and explanation were repeated for each step so that the children could individually follow and prepare their own recipe simultaneously. Afterward, children were invited to taste their recipe and have a group discussion on healthy eating • In the intervention group, children participated in three cooking workshops that included three recipes. One workshop was held per week for three weeks. The comparison groups 1–3 conducted one workshop	• Cooking skills: ∅ • Nutrition knowledge: ⬆ (intervention group and comparison group 3)	NA
Kimiecik et al., 2021 [18]	• 35 campers: 13- 15 years old • 9 completed qualitative interviews • 1 camp	• The Learning in Fitness and Education through Sports (LiFEsports) (PA) • Over four weeks, nine sport-based and healthy lifestyle activities (e.g., soccer, basketball) led by trained recreational sports leaders for four hours each day + daily classroom-based social skills curriculum called “Chalk Talk” for one hour • Staff and older youth encourage younger youth to reflect on their use of S.E.T.S. during the camp and ask youth to verbalize ways to transfer each skill to other areas of their lives at the end of every sports session	• Healthy lifestyles, social competence, and social sports experience: ∅ (⬆ non- significant) • Healthy lifestyles: ⬆ (Girls)	NA
Lawman et al., 2019 [30]	• 2 586 Campers: 3–19 years old • 28 camps	• The Hydrate Philly Intervention (HE) • Seven to nine months • Replacing old and unappealing water fountains with appealing water-bottle- filling “hydration stations” (one or two per site) • Distribution of reusable water bottles to each camper and some staff • A campaign to promote the acceptability of tap water, brief training for recreation center staff, a game the sites could use to encourage water consumption, and parent handouts was implemented at intervention sites • Staff training included behavioral and social strategies for staff to discourage SSB consumption and encourage water consumption • Half-page flyers in English and Spanish were distributed to families of youth attending summer programming at sites	• Water use: ⬆ • Reusable bottle counts: ⬆ • Youth carrying SSBs at camp: ∅ • Staff’s past 30-day SSB consumption frequency: ⬇ • Maintenance problems: ⬇ (trend)	NA
Mabary-Olsen et al., 2015 [31]	• 74 campers: 9- 14 years old & 16–18 years old • 2 camps	• Wellness Camp—Summer 4-H camps (HE) • Three weeks • Intervention campers received two to three hours of experiential learning in gardening, culinary, and nutrition each morning • Each experience included a hands-on learning activity followed by a discussion to reflect (share and process) and apply (generalize and apply) their observations to similar/different situations • Intervention weeks also had lunch menus tailored to incorporate vegetables harvested from the garden and prepared during the culinary lessons (i.e., homemade salsa for tacos) • Youth in the intervention also received a take-home kit intended to influence the home environment	• Nutrition knowledge: ⬆ (trend) (from baseline to 6 months post-camp) • Most preferred home environment: ⬆ (trend between the control and intervention at 6 months post-camp) • Most and least preferred home food environment: ⬆ (intervention) • Campers' self-efficacy and overall FV preferences: ⬆ (trend)(intervention) • Consumption of spinach and bell peppers: ⬆ (trend) • Consumption of zucchini: ⬆	NA
Maxwell et al., 2018 [32]	• 50 campers • 1 camp	• Eating Veggies Is Fun! (HE) • Daily for two weeks • The intervention consisted of repeated tasting only of the initially disliked vegetables (i.e., Jicama, red bell pepper, mushroom, zucchini, and sugar snap pea) because the fruits were uniformly liked • Plates with small pieces of these five initially disliked target vegetables were offered to all participating children in a group setting	• Liking the 5 targeted vegetables: ⬆ • Liking the 7 nontargeted vegetables: ∅ • Liking jicama: ⬆ • Liking the nontargeted vegetable celery: ⬇ • Liking to try new foods and accessibility to and consumption of FV “yesterday”: ∅ • Liking any of the vegetables examined singly and how much children reported liking to try new foods: ∅ • Consumption of fruits and vegetables “yesterday”: ∅	NA
Murad et al., 2021 [33]	• 17 campers • 1 camp	• Farm to Future (HE) • One week on Google Meets • Develop cooking skills, provide hands-on experience cooking simple meals and snacks, educate about a balanced diet, educate about sustainable cooking and eating, and provide daily physical activities • Included a daily nutrition or cooking lesson (i.e., basic nutrition topics such as food and knife safety, fermentation, and dairy foods), preparation of both a lunch and afternoon snack recipe, one or two recorded physical activity sessions, and a cooking activity to demonstrate food science principles • Parents were advised to be close by to help	• Better at cooking: ⬆ • Better at trying new foods: ⬆ • Confident they can make more sustainable food choices: ⬆ • Food literacy: ∅ (nine pre- and post-survey) • Most participants reported liking interacting with other children and being able to cook a real meal, not just desserts, to feed themselves and their families	NA
Reverter-Masia et al., 2012 [19]	• 102 campers • 1 camp	• Healthy lifestyle guide pyramid (PA + HE) • Two sessions of 50 min (presentation, debate, and conclusions) • The pyramid has faces oriented towards achieving goal; daily food intake, daily activities, traditional food guide pyramid adapted to children’s and adolescents’ energy, nutritional and hydration needs, daily and lifelong habits, and health • The role of the instructor varied depending on the stage of each session: describing the pyramid, arguing, and explaining its contents, moderating and dynamizing the debates, focusing one’s attention on specific aspects, asking questions and conceptualizing answers • All children were handed out photocopies of the “Healthy lifestyle guide pyramid” to talk about it with their parents	• Intake of whole milk, cold meats, and sweet things: ⬇ • Fruit and cereal consumption: ⬆ (after the first intervention, not maintained) • Consumption of butter and nuts: ∅ • Most participants reported liking interacting with other children and being able to cook a real meal to feed themselves and their families • Physical activity: ⬆ (short-and long-term) • Hours of television exposure: ⬇ (both groups, long-term)	NA
Seal & Seal 2011 [34]	• 18 campers: 8- 12 years old • 10 camps	• Wellness Summer Camp (WSC) (PA + HE) • 10 days, from 8 am to 4 pm • Trained camp counsellors worked with the children in small groups (four to five children per group); therefore, each child received age-appropriate interventions and individualized attention • The PA: physical education that promoted lifelong PA • The nutrition: nutrition education (emphasized a diet rich in vegetables, fruits, unsaturated fats, and whole grains and low in saturated fat and sugar)	• Nutrition knowledge (i.e., healthy foods and healthy snacks): ⬆ • Knowledge of physical activity: ⬆ (Short- term positive effects) • Eating behaviors: ⬆ (Short-term positive effects) • PA: ∅ • Self-perception of competence: ⬆ (Short-term positive effects)	NA
Tauriello et al., 2020 [35]	• 23 campers: 6–8 years old • 1 camp	• (HE) • 1 h, taste exposures during recurrent morning programming + participation in a series of three group games • The repeated exposure classroom received only individual taste exposures to their target vegetable	• Preferences for target vegetables: ⬆ (both groups)	NA
Tilley et al., 2014 [36]	• 1977 campers • 241 counsellors • 4 camps	• The “Healthy-Lunchbox- Challenge” (HE) • 11-week SDC program • Parent and staff education: Healthy eating education materials included a description of the HLC mission and procedures, a “Building a Better Lunchbox” guide • Child and staff incentive program to influence parental decisions of foods and beverages purchased for SDC. Points were tallied by SDC staff. Prizes were awarded to groups with the highest points at the end of each week	• FV and water brought to SDC: ⬆ • Chips and non-100% fruit juices brought to SDC: ⬇	• FV brought to SDC by staff: ⬆ • Water brought to SDC by staff: ⬇ (not statistically significant) • Chips brought to SDC by staff: ⬇ • Soda brought to SDC by staff: ⬇ (trend)
Warner et al., 2021 [37]	• 45 campers: 6- 10 years old • 30 counsellors • 1 camp	• Maple Leaf Sport and Entertainment LaunchPad (PA) • Two weeks (nine days), from 8:30 am to 4:00 PM • Program was delivered at a large SFD facility that offers free programming to youth facing barriers to a positive development • Used fundamental of movement skills (FMS) activities, sport-specific activities, and games of low organization to develop physical literacy. Rotations of activities including supervised free play, snack-times, low-organization games, active play in small groups, and sports	• Overall FMS: ⬆ (boys > girls) • Self-perceptions of PL (competence, confidence, motivation, and knowledge): ⬆ (boys > girls)	• A high staff-to-youth ratio with well-trained, caring leaders ensured a consistent presence of nurturing adults • The inclusion of "Leaders in Training" as part of the staff team provided an element of peer mentoring to youth participants, who saw themselves reflected in the demographics of these staff
Weaver, Beets, Saunders et al., 2014 [38]	• ~ 800 campers daily: under 12 years old • ~ 300 counsellors • 4 camps	• (PA) • Four days a week/eight weeks • Professional development training, workshops, and weekly feedback and self- evaluation • Six on-site booster trainings (reinforce HEPA promotion strategies and principles LET US Play covered in the 5Ms trainings) • SDCs were structured with a variety of activities including free-play opportunities; organized games, water-based activities, and enrichment activities such as arts & crafts	• % of children physically active: ⬆ (boys, during overall PA opportunities/ girls, during organized activities) • % of children sedentary: ⬇ (boys and girls, especially during organized activity) • Not all changes reached statistical significance (sedentary behavior and MVPA depending on the school level)	• HEPA promoting staff behaviors: ⬆ • HEPA discouraging staff behaviors: ⬇
Weaver, Beets, Turner-McGrievy et al., 2014 [39]	• 600 campers • 120 counsellors • 4 camps	• (PA) • Four days a week/eight weeks • A daylong (eight hours) training occurred each year in May followed by a PA training session which lasted approximately 90 min • A workshop on schedule modification and weekly feedback from the evaluation team were also offered • Nine, two-hour on-site booster training sessions were offered in the two intervention summers (6x/2012 and 4x/2013) (real-time feedback and suggestions aligned with the training focusing on modifying games to enhance child PA, managing PA environments effectively, and modeling and encouraging child PA)	• % of sedentary children: ⬇ • % boys engaged in MVPA: ⬆ • % girls engaged in MVPA: ⬆ • All these changes in MVPA reached statistical significance except for the children in grades 4 and 5	• Promoting children's PA: ⬆
Weaver et al., 2017 [40]	• 1 830 campers: 5–12 years old • 20 camps	• Turn up the healthy eating and activity time (HEAT) (PA) • Five days a week/eight weeks • Camp leaders and staff receive training to expand, extend, and enhance PA opportunities (i.e., a single 90-min professional development training session and a 30-min discussion on strategies to address challenges observed with increasing children's PA) • Two on-site booster sessions (Walkthrough of the SDC and discussion to address challenges observed with increasing children’s MVPA)	• Campers meeting the 60 min/day MVPA guideline: ⬆	• Results indicate that the STEPs intervention SDCs were successful in extending and enhancing PA opportunities compared to control SDCs • Yet, there was no evidence to suggest they expand PA opportunities when compared to control SDCs
Werner et al., 2012 [41]	• 760 campers: 6- 9 years old	• Active Generations (PA + SB + HE) • Ten lessons • Obesity prevention program with a focus on nutrition education and PA • Utilizes older adult volunteers to implement the program meant to increase PA participation, inform on nutrition and food labels, and decrease sedentary time	• FV consumption post-program: ⬆ • Nutrition knowledge: ⬆ • Likely to read food labels: ⬆ • Confidence in participating in PA: ⬆ • Daily screen time: ⬇	NA
Williams et al., 2019 [42]	• 15 campers: 7- 15 years old • 1 camp	• Child-focused cooking curriculum (HE) • Daily, during seven weeks • Twice per day, a 10-min lesson on a given recipe • Recipes were chosen to fit the balanced plate and cover general nutrition topics in an age-appropriate manner • Staff would assist in preparation for younger groups, and let older groups create the recipe with minimal assistance. While they ate, leaders reviewed key aspects of the recipe that were healthy and how it fits into the balanced plate	• Children overwhelmingly enjoyed the cooking camp and discussed it extensively with their parents at home • Almost all tried to replicate recipes at home	NA
Wilson et al., 2017 [43]	• 88 campers: 5- 11 years old, mean age of 7.8 years old • 1 camp	• Goal setting at summer camp (PA) • Four weeks • The goal-setting programs differed each week; campers set individual goals, small group goals, and then a camp-wide goal • Individual goal; each child set their own step count goal • Group goal; campers are placed into small groups every week at camp led by a counsellor. Each group set a collective group step count goal • Camp-wide goal; the entire camp set a collective step count goal • Feedback was provided at the end of every camp day based on goal setting	• Step counts: ⬆ (individual and camp-wide goal setting) • Enjoyment: ⬆ (group and camp-wide goal setting) • Boys found to be more physically active than girls • Older campers enjoyed PA less	NA

The five studies that measured the determinants of physical activity observed increases following the interventions. Specifically, Gachupin et al. [17], Seal & Seal [34], and Werner et al. [41] measured increases in knowledge (e.g., how long they should be active each day and places where they can be active), Anderson-Butcher et al. [16], Seal & Seal [34], and Werner et al. [41] measured increases in perception of control (i.e., self-efficacy or self-perception of competence) while Gachupin et al. [17] and Wilson et al. [43] measured increases in positive attitude and enjoyment, respectively.

Only one intervention directly targeted sedentary behaviors, but a few studies whose intervention targeted physical activity still measured sedentary behaviors. Although few studies have ultimately measured sedentary behaviors, all those that have measured them have observed positive changes. In all cases, screen-time or television time [19, 24, 41] and sedentary time [38, 39] decreased during the summer or following the intervention.

### Effects of Summer Day Camp interventions targeting healthy eating

Nine of the twelve studies that measured eating habits observed positive changes. Studies by Baranowski et al. [21], Beets et al. [23], Bohnert et al. [24], Mabary-Olsen et al. [31], Reverter-Masia et al. [19], Seal & Seal [34], Tilley et al. [36], and Werner et al. [41] measured increases in fruit and/or vegetable (FV) consumption and Baranowski et al. [21], Lawman et al. [30], and Tilley et al. [36] measured an increase in water consumption. These same studies measured a decrease in the consumption of sugary drinks among campers and counsellors [21, 30, 36]. Some studies such as Beets et al. [23], Reverter-Masia et al. [19], and Tilley et al. [36] also measured decreases in unhealthy behaviors (e.g., soda/pop, non-100% juice, chips, and fast food). Finally, some isolated studies measured a decrease in energy intake from lipids [21] or a decrease in the consumption of dairy products [19, 24].

Thirteen of the fifteen studies that measured the determinants of healthy eating observed increases following the interventions. Specifically, Beets et al. [22], Condrasky et al. [26], Gachupin et al. [17], Jacob et al. [20], Mabary-Olsen et al. [31], Seal & Seal [34], and Werner et al. [41] measured an increase in food-related knowledge. Beets et al. [22], Condrasky et al. [26], Harmon et al. [28], Mabary-Olsen et al. [31], Murad et al. [33], and Seal & Seal [34] measured increases in perceived control (i.e., self-efficacy or self-perception of competence towards cooking or healthy eating) while Beets et al. [22], Gachupin et al. [17], and Harmon et al. [28] measured favorable changes in attitudes towards healthy foods. Finally, Ehrenberg et al. [27], Heim et al. [29], Mabary-Olsen et al. [31], Tauriello et al. [35], and Maxwell et al. [32] measured increases in preferences towards fruits or vegetables, and Werner et al. [41] measured increases in label reading.

## Discussion

Children and young adolescents must meet the recommendations for physical activity, sedentary behavior, and healthy eating throughout the year for optimal health. SDCs have the potential to replace the organization of school settings during summer breaks, but few interventions have been conducted in SDCs. In this review, we synthesized and summarized interventions that have integrated physical activity, sedentary behaviors, and healthy eating promotion in SDCs to identify some key lessons for future programs on healthy lifestyles targeting children and young adolescents. Our results showed that the number of studies targeting physical activity and healthy eating in SDCs was relatively low. We found that eight of the twenty-eight studies meeting eligibility criteria were limited to promoting physical activity, fourteen were limited to healthy eating promotion and five included both. Additionally, only one intervention targeted sedentary behaviors.

Physical activity, sedentary behaviors, and healthy eating are behaviors influenced by individual and environmental factors. The articles in this review are primarily focused on the behaviors themselves or their determinants. Fourteen studies included the promotion of physical activity in their intervention and most of them indicated positive changes in physical activity (i.e., MVPA, perceived behaviors, and number of steps) and/or their determinants (i.e., knowledge, perception of control, and attitude) [16–19, 24, 34, 38–41, 43]. Positive effects of physical activity promotion on sedentary behaviors (i.e., screen-time, television time, and sedentary time) were also observed in five studies [19, 24, 38, 39, 41]. For interventions that promoted healthy eating, most of them reported positive changes (i.e., FV, water, and SSB consumption) and/or their determinants (i.e., food-related knowledge, control perception, attitudes, and preferences) [17, 19–24, 26–36, 41]. Overall, only three of fourteen studies that measured physical activity [21, 25, 37] and one of twenty studies [42] that measured healthy eating did not observe changes in the target behavior or their determinants. Several methodological factors may explain these results, such as the specific content of the intervention, and the involvement of counsellors and/or parents.

Among all the factors that can explain success in intervention, the use of a theoretical frame to build the intervention represents an important aspect. Indeed, the use of a theory, often a theory of behavior change, is associated with a greater rate of success when it comes to promoting healthy lifestyle behaviors [44]. Most interventions targeting physical activity, except for four studies [16–19], were based on theories and all except three interventions targeting healthy eating [17, 19, 20] relied on a theoretical framework. The concepts of social cognitive theory and the ecological model of human development were the main elements used in the design of the interventions. Surprisingly, the results of the studies in this review do not differ according to the use of a theory.

Our results highlight a variety of different types of strategies that influenced physical activity, sedentary behaviors, and healthy eating. The most efficient strategies identified were goal setting or point systems, modifications of physical environments, physical activity education activities, promotion of physical activity with counsellors, cooking workshops or specific healthy eating education, and activities on overall healthy lifestyle behaviors including sedentary behaviors. The interventions could contain several strategies, but the majority had only one. Among those strategies, using goal setting seems promising. For instance, Wilson et al. [43] show that an intervention focusing primarily on goal setting to promote physical activity generally increases the number of steps taken and enjoyment of physical activity in SDCs. After a week, setting individual and camp-wide goals increased the number of steps. Conversely, group goal setting (vs. individual) did not affect step count but still had a positive influence on camper enjoyment. As for healthy eating, Baranowski et al. [21] and Heim et al. [29] used goal setting as a secondary component of the intervention. Even if both interventions had positive effects on the behavior, it is difficult to conclude if this specific strategy is responsible for the changes. Similarly, an intervention evaluated by Beets et al. [23] and Tilley et al. [36] used a point system to encourage healthy eating among campers. In both cases, the authors observed increases in FV consumption and a decrease in unhealthy behaviors in both campers and counsellors. Adding goal setting or point system, therefore, seems to be a very interesting component for interventions promoting physical activity and healthy eating in SDCs, mainly when it comes to individual and camp-wide goals.

Although physical environments are very important in the adoption of healthy behaviors [45], only three studies changed the physical environment in the camp to influence eating habits [29–31]. Accordingly, Lawman et al. [30] replaced old and unappealing water fountains (i.e., one or two per site) and distributed reusable water bottles to campers and some staff [30]. In addition, they ran a campaign to promote the acceptability of tap water, including a brief training for staff based on behavioral and social strategies which aimed to discourage SSB consumption and encourage water consumption [30]. Results indicate that at the end of the camp, campers consumed more water, there were more reusable bottles on the sites and the staff consumption of sugar-sweetened beverages over the last 30 days decreased. The other two studies changed physical environments by creating gardens and this had positive effects on young people. Indeed, Heim et al. [29] measured an increase in preference for vegetables, and Mabary-Olsen et al. [31] measured tendencies towards an increase in knowledge and self-efficacy towards vegetables. Thus, modification of the physical environments in the camps combined with a social campaign represents another interesting strategy to promote healthy behaviors.

The most common strategy used in physical activity interventions is education implemented using direct or indirect strategies. Some interventions included physical activity education directly to campers [16, 18, 37] and while others, rather included physical activity education through counsellors [25, 38–40]. Anderson-Butcher et al. [16], Kimiecik et al. [18], and Warner et al. [37] have all respectively evaluated positive changes following direct education on either self-efficacy, girls' healthy behaviors, or physical literacy. In the case of the intervention evaluated by Brazendale et al. [25] and Weaver et al. [40], based on the theory of expanded, extended, and enhanced opportunities which include indirect education through counsellors as the main strategy, the authors report an increase in the number of campers meeting the recommendations of 60 min of PA per day after the first year [40]. The results after four years also show that there is no difference between one year and two years of intervention. However, even though campers are ultimately no more likely to meet the recommendations of 60 min of PA per day at the end of the study, girls and boys were still 3.5 and 3.7 times more likely to meet the 60 min/d guidelines during intervention summers versus follow-up, respectively [25]. As for the intervention evaluated by Weaver, Beets, Saunders et al. [38] and Weaver, Beets, Turner-McGrievy et al. [39], it is rather based on the Let Us Play theory which also aimed to use an indirect education strategy. These studies have both measured increases in MVPA and a decrease in sedentary behavior in some campers. Interventions including education, therefore, have positive effects on physical activity of campers both when it is addressed directly to them or when it is implemented indirectly via the counsellors. Moreover, targeting counsellors makes it possible not only to target campers but also young adolescents.

The promotion of healthy eating is also essentially done through education with culinary workshops [20, 22, 26–28, 31, 33, 42]. Culinary workshops in SDCs improve the determinants of healthy eating, such as knowledge [20, 22, 26] preference [27], and the perception of control [22, 28, 33]. However, they have less effect on healthy eating whereas only one study observed an effect on campers' eating habits [31] and one study reported that campers replicated recipes learned in the workshops [28]. Other interventions have instead used repeated exposure to influence healthy eating among campers [32, 35]. Both studies improved campers' preferences/liking for vegetables, yet it is not known if this influenced their eating habits. Although it is difficult to conclude that cooking workshops in SDCs influence the eating habits of children and young adolescents, it has been shown that few cooking workshop opportunities are enough to improve the determinants of behavior change towards healthy eating [26].

While some interventions targeted a single behavior, some interventions targeted multiple behaviors (i.e., physical activity, sedentary behaviors, and healthy eating) altogether [17, 19, 21, 24, 34, 41]. Although the six interventions used education to improve campers' lifestyle behaviors, certain particularities such as the use of a web program in addition to the camp experience [21], six hours of totally structured activity [24], the use of the traditional food guide pyramid adapted to children's and adolescents to create discussions [19], and the inclusion of sedentary behaviors [41] distinguish them. The six studies all reported positive changes in behaviors or determinants, but they did not necessarily impact all the behaviors. For instance, the study of Baranowski et al. [21] influenced the eating habits of campers, but it did not modify the physical activity of the girls in the program. One of the reasons that may explain the lack of change is the low connection rate of participants to the web program. Similarly, Seal & Seal [34] also measured changes in participants' eating habits following the intervention, but there was no change in physical activity per se despite an increase in knowledge and self-perception. In sum, interventions that simultaneously target several lifestyle behaviors have reported positive effects on one or more behaviors and determinants of all the targeted behaviors.

The involvement of counsellors in the implementation of the interventions represents an interesting resource for promoting healthy lifestyle behaviors as they are in contact with campers daily. A total of eight interventions presented in eleven studies included counsellors in different ways to promote physical activity or healthy eating [18, 23, 25, 29, 30, 34, 36, 38–40, 43] but the implementation is not always well described. Three interventions presented in six studies put more emphasis on the role of counsellors; these interventions mainly consisted of personalized training and supporting counsellors and camps with booster sessions (i.e., visits or communications with the camp to ensure the proper implementation of the intervention) [23, 25, 36, 38–40]. The major difference between these interventions, in addition to the behavior promoted, is the content of the interventions and the number of booster sessions The intervention by Weaver, Beets, Turner-McGrievy et al. [39] and Weaver, Beets, Saunders et al. [38] contains additional elements for counsellors such as training to help use template schedules and six booster sessions. These sessions were in the camps where the program staff provided oral feedback based on weekly assessments. Beets et al. [23] and Tilley et al. [36] also include personalized training to counsellors and booster sessions (i.e., weekly communications and site visits) by the program staff to ensure that the program was properly implemented. Brazendale et al. [25] and Weaver et al. [40] include personalized training to counsellors based on the theory to expand, extend, and enhance PA opportunities and two on-site booster sessions for counsellors during the summer. Results from the review indicate that the two of these interventions had a positive effect on physical activity and eating habits. Overall, it seems that the intervention with individualized training to counsellors and more support for the camps in the implementation (i.e., booster sessions) have a better impact on the promotion of healthy lifestyle behaviors and ultimately on the behaviors of campers and counsellors.

Several interventions involved the parents of participating campers in physical activity [17, 19] and healthy eating promotion [19, 21, 23, 28, 30, 36]. All interventions that included parents did so by educating them to sustain behaviors promoted in camps at home, but two interventions were also asking parents to help campers in activities that had to be done at home [21, 28], and two interventions also used a point system at camp and campers’ rewards to further incentivize parents to modify camper lunch boxes [23, 36]. Concretely, it is difficult to say whether the inclusion of parents is effective since only one measured their commitment and it was rather weak. However, Anderson-Butcher et al. [16] aimed to assess the influence of parental support on physical activity determinants and found that parental support was a predictor of self-efficacy and intention toward healthy behaviors [16]. Additionally, the authors observed that parental support increased the beneficial effect of support from staff on self-efficacy and intention toward healthy behaviors. The inclusion of parents, therefore, seems interesting for campers’ behaviors, but also to increase the influence and support of counsellors on them.

### Strengths and limitations

The main strength of this review is the extensive article search strategies with syntax that made it possible to include as many articles as possible on several platforms. Another strength is the inclusion of interventions targeting sedentary behaviors as most of the reviews on healthy lifestyle interventions in after-school programs included physical activity and healthy eating but not sedentary behaviors. As day camps represent an important setting that can contribute to reduce sedentary behaviors, the identification of strategies that may contribute reducing sedentary behaviors represents an important step forward in this context. The use of PRISMA-ScR, is also a strength of this scoping review. This review is, however, also subject to some limitations. In line with scoping review objectives (i.e., more descriptive review compared to systematic reviews), the quality of the articles was not evaluated. Even if the quality of the articles had not been assessed, the present scooping review highlighted that most studies were cross-sectional and had a quasi-experimental design, mainly because they did not include a control group, which can both have effects on the capacity to infer causation. Also, gray literature was not included in this review. Moreover, the lack of details on training and the degree of intervention implementation compromises comparisons between programs and influences the conclusions of this review. Finally, the length of the evaluation was short, one summer for most interventions, and the studies that made several evaluations did not necessarily assess the same campers across the years.

## Conclusion

This scoping review revealed that the targeted behaviors such as physical activity, sedentary behaviors, eating habits, and their determinants significantly improved in most intervention studies. Considering that physical activity, sedentary behaviors, and eating habits are not optimal in children and young adolescents during the summer and even in the SDCs, promoting healthy behaviors during this specific period is needed. This review highlights that strategies such as goal setting or a point system, modification of the physical environments (e.g., garden) combined with a social campaign, the inclusion of counsellors, comprehensive and individualized counsellor training, multiple booster sessions, and parents’ support are key elements for the success of an intervention promoting healthy lifestyle behaviors in SDCs. Future research should include more long-term intervention studies including a control group to better assess the causality between the promotion of healthy behaviors in SDCs and the effects on camper’s behavior. Finally, the most important recommendation of this review is to make interventions that will not only target physical activity and healthy eating but also sedentary behaviors to develop more global lifestyle interventions.

## Supplementary Information

**Additional file 1.**

## Data Availability

The datasets used and/or analysed during the current study are available from the corresponding author on reasonable request.

## Abbreviations

SDCs: Summer day camps
MVPA: Moderate-to-vigorous physical activity
SSB: Sugar-sweetened beverages
FV: Fruit and vegetable
PA: Physical activity
BCT: Behavioral choice theory
SCT: Social cognitive theory
STEPs: Strategies to enhance practices
TEO: Theory of expanded, extended, and enhanced opportunities
