# Supplementary material for: Interventions to promote healthy lifestyle behaviors in children and adolescents in summer day camps: a scoping review
Source: BMC Public Health. 2023 Apr 26;23:773. doi: 10.1186/s12889-023-15521-1 (PMC10134537; doi:10.1186/s12889-023-15521-1)
Supplement: Supplementary file 1 — Additional file 1. [file 12889_2023_15521_MOESM1_ESM.docx]

### Additional file 1

### Detailed database:

### EBSCOhost:

1. **SPORTDISCUS**

Database unlimited

Period of coverage: 1930 – present (full text)

Update Frequency: Monthly

1. **CHILD DEVELOPMENT & ADOLESCENT STUDIES**

Database unlimited

Period of coverage: 1927 – present (full text)

Update Frequency: Monthly

1. **ERIC (EBSCO)**

Database unlimited

Period of coverage: 1966 – present (full text)

Update Frequency: Monthly

1. **EDUCATION SOURCE (EBSCO)**

Database unlimited

Period of coverage: 1927 – present (full text)

Update Frequency: Monthly

1. **OVID**
2. **MEDLINE**

Database unlimited

Period of coverage: 1946 – present (full text)

Update Frequency: Daily

1. **EMBASE**

Database unlimited

Period of coverage: 1966 – present (full text)

Update Frequency: Daily

1. **CLAVIRATE**
2. **Web of Science**

Database: Core collection

- Emerging Sources Citation Index (ESCI) --2005-present
- Science Citation Index Expanded (SCI-EXPANDED) --1900-present
- Social Sciences Citation Index (SSCI) --1900-present
- Arts & Humanities Citation Index (A&HCI) --1975-present

Update Frequency: Daily
